# Comparing prevalence of chronic kidney disease and its risk factors between population-based surveys in Russia and Norway

**DOI:** 10.1186/s12882-022-02738-2

**Published:** 2022-04-14

**Authors:** Sarah Cook, Marit D. Solbu, Anne Elise Eggen, Olena Iakunchykova, Maria Averina, Laila A. Hopstock, Kamila Kholmatova, Alexander V. Kudryavtsev, David A. Leon, Sofia Malyutina, Andrew Ryabikov, Elizabeth Williamson, Dorothea Nitsch

**Affiliations:** 1 Faculty of Epidemiology and Population Health, London School of Hygiene & Tropical Medicine, London, UK; 2 National Heart and Lung Institute, Imperial College London, London, UK; 3 Metabolic and Renal Research Group, UiT The Arctic University of Norway, Tromsø, Norway; 4 Section of Nephrology, Division of Internal Medicine, University Hospital of North Norway, Tromsø, Norway; 5 Department of Community Medicine, UiT The Arctic University of Norway, Tromsø, Norway; 6 Department of Laboratory Medicine, University Hospital of North Norway, Tromsø, Norway; 7 Northern State Medical University, Arkhangelsk, Russian Federation; 8 International Laboratory for Population and Health, National Research University Higher School of Economics, Moscow, Russian Federation; 9 Research Institute of Internal and Preventive Medicine, Branch of Institute of Cytology and Genetics, Siberian Branch of the Russian Academy of Sciences, Novosibirsk, Russian Federation; 10 Novosibirsk State Medical University, Russian Ministry of Health, Novosibirsk, Russian Federation

**Keywords:** Chronic kidney disease, Epidemiology, Norway, Risk factors, Russian Federation

## Abstract

**Background:**

Little data exists on the prevalence of chronic kidney disease (CKD) in the Russian population. We aimed to estimate the prevalence of CKD in a population-based study in Russia, compare with a similar study in Norway, and investigate whether differences in risk factors explained between-study differences in CKD.

**Methods:**

We compared age- and sex-standardised prevalence of reduced eGFR (< 60 ml/min/1.73m^2^ CKD-EPI creatinine equation), albuminuria and or a composite indicator of CKD (one measure of either reduced eGFR or albuminuria) between participants aged 40–69 in the population-based Know Your Heart (KYH) study, Russia (2015–2018 *N* = 4607) and the seventh Tromsø Study (Tromsø7), Norway (2015–2016 *N* = 17,646). We assessed the contribution of established CKD risk factors (low education, diabetes, hypertension, antihypertensive use, smoking, obesity) to between-study differences using logistic regression.

**Results:**

Prevalence of reduced eGFR or albuminuria was 6.5% (95% Confidence Interval (CI) 5.4, 7.7) in KYH and 4.6% (95% CI 4.0, 5.2) in Tromsø7 standardised for sex and age. Odds of both clinical outcomes were higher in KYH than Tromsø7 (reduced eGFR OR 2.06 95% CI 1.67, 2.54; albuminuria OR 1.54 95% CI 1.16, 2.03) adjusted for sex and age. Risk factor adjustment explained the observed between-study difference in albuminuria (OR 0.92 95% CI 0.68, 1.25) but only partially reduced eGFR (OR 1.42 95% CI 1.11, 1.82). The strongest explanatory factors for the between-study difference was higher use of antihypertensives (Russian sample) for reduced eGFR and mean diastolic blood pressure for albuminuria.

**Conclusions:**

We found evidence of a higher burden of CKD within the sample from the population in Arkhangelsk and Novosibirsk compared to Tromsø, partly explained by between-study population differences in established risk factors. In particular hypertension defined by medication use was an important factor associated with the higher CKD prevalence in the Russian sample.

**Supplementary Information:**

The online version contains supplementary material available at 10.1186/s12882-022-02738-2.

## Background

Chronic kidney disease (CKD) is an increasingly important public health problem associated with high morbidity and mortality. The prevalence of CKD varies widely around the world ranging from 3 to 17% with prevalence increasing sharply with age [1–4].

Cardiovascular disease (CVD) mortality in Russia is very high [5, 6], four times higher than in neighbouring Norway in 2015 [6, 7]. CKD is strongly related to CVD [8–11]. Recent population studies in Russia have shown a high prevalence of risk factors for CKD - hypertension [12, 13], diabetes [14–17] and obesity [12, 18] - all indicating the burden of CKD is also likely to be high. One population-based study from the city of Novosibirsk (2013–2016) has investigated estimated glomerular filtration rate (eGFR) among 1064 adults aged 25–45 [19]. In this sample mildly reduced kidney function (eGFR< 90 ml/min/1.73m^2^) was found in 9.8% of men and 34% of women [19]. The prevalence of CKD (eGFR< 60 ml/min/1.73m^2^) was very low (0.3% of total sample) although this was not surprising because of the relatively young age-profile of the study population. Quantifying the burden of CKD in older adults is important and placing this in the context of other populations is likely to be informative. In particular it is important to know the burden of disease and relative contribution of risk factors in mid-life when kidney damage is in the early stages and there is a key role for health policy intervention in reducing the burden of disease in older adults.

The aim of this paper is to describe the comparative prevalence of CKD among participants in population-based surveys in Russia and Norway, a neighbouring country with substantially different CVD and all-cause mortality rates, and the extent to which differences in the distribution of established risk factors explain any observed between-country differences in the prevalence of CKD among men and women aged 40–69 years old.

## Methods

The study population were participants aged 40–69 years taking part in two population-based surveys, the Know Your Heart (KYH) study, Russia (2015–2018), and the seventh wave of the Tromsø Study (Tromsø7), Norway (2015–2016). These studies were conducted in parallel as part of the Heart to Heart project aimed at understanding the reasons for Russia having much higher rates of CVD mortality than Norway. Several aspects of data collection between the studies have been harmonized (including calibration of laboratory tests of blood samples) [20] providing a unique opportunity to compare levels of CKD in the general population of both countries.

### Know your heart (Russia)

KYH is a cross-sectional study including 4607 men and women aged 35–69 years recruited from the general population in the Russian cities of Arkhangelsk and Novosibirsk, described in detail previously [21]. The response rate in the study was higher for the city of Arkhangelsk (43.9% of participants 35–69 out of all addresses sampled completed a baseline interview compared to 20.9% from Novosibirsk) [21].

**Fig. 1:**
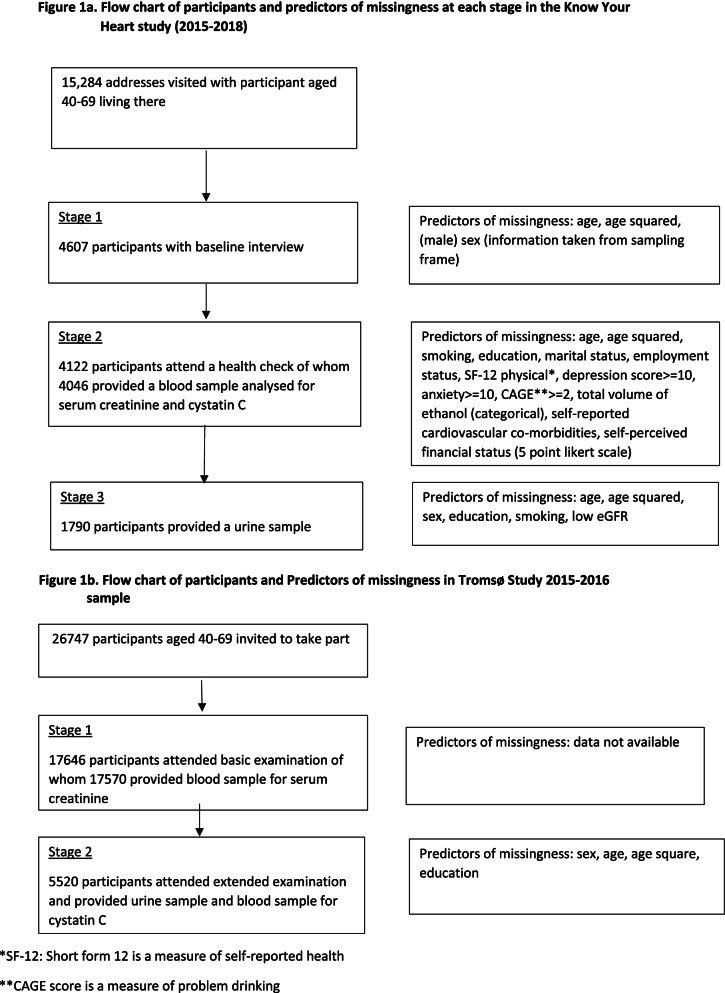
Flow Chart of Participants and predictors of missingness. **a** Flow chart of participants and predictors of missingness at each stage in the Know Your Heart study (2015–2018). **b** Flow chart of participants and Predictors of missingness in Tromsø Study (2015–2016)

In brief, a random sample of addresses where a person aged 35–69 years was living, stratified by age, sex and district was selected from a population register. Addresses were visited by trained interviewers who recruited participants to take part in the study. Participants who agreed to take part completed an interview about their health (stage 1) and were invited to attend a health check at a polyclinic (stage 2). At stage 2 all participants were asked to provide a blood sample and a spot urine sample. Participants had the choice to provide a urine sample during the health check or to do this at home and return it to the clinic. Participation at each stage of the study is shown in Fig. 1a.

Blood samples were collected in SST II vacutainers BD® (Beckton, Dickinson and Company, Preanalytical Systems, US). Samples were left at room temperature for 30 min and then stored at 4 °C before processing. Samples were centrifuged in cooled centrifuges at 4 °C at 2100-2200 g for 15 min. Urine sample were collected using Beckton Dickinson urine collection pots. If participants did this at home they were instructed to store the sample at 4 °C and return to the clinic within 18 h in order to meet target of freezing samples within 24 h. Blood samples were frozen within 2 h and urine samples within 24 h at -20 °C, and transferred to a − 80 °C freezer within 3 weeks. All samples were analysed centrally in one batch at the end of the study at the same central laboratory in Moscow. Serum creatinine was measured using an uncompensated kinetic Jaffe reaction on a Beckman Coulter machine. Serum cystatin C was assessed with an enzymatic method using Particle enhanced Immunoturbidimetric test. Urinary albumin was measured using Immuno-turbidimetric test and urine creatinine was traceable to the IDMS method using a compensated kinetic Jaffe reaction on a Beckman Coulter machine. Coefficient of analytical variation (CV) For serum creatinine CV was < 7%, for serum cystatin C CV was < 4%, for urinary albumin CV was < 3% and for urinary creatinine CV was < 9%.

### Tromsø7 (Norway)

The Tromsø Study [22] is an ongoing population-based study of residents of the Norwegian municipality Tromsø. In Tromsø7, all residents aged 40 years and older were invited, of which 21,083 men and women participated (65%), and 17,646 were aged 40–69 years.

All participants were invited to a basic examination including questionnaires, biological sampling (including serum creatinine) and medical examinations. A random subset was invited to an extended clinical examination with additional biological sampling (including serum cystatin C and urine samples). Participation at each stage of the study is shown in Fig. 1b.

Blood samples were collected in SST tubes and left for 30 min at room temperature and then centrifuged within 1 h for 10 min at 2000 g. Analyses were done within the same day as sample collection at the University Hospital of North Northway laboratory that is accredited according to the ISO 15189 standard.

Urine samples were first morning void samples taken on three consecutive days. Participants returned the samples when they attended the second part of the examination and analyses were done on the same. For comparison with KYH the first day sample was used.

Serum creatinine was measured using enzymatic colorimetric method traceable to isotope dilution mass spectrometry, and serum cystatin C using the immunoturbimetric method. Urinary albumin was measured using colorimetric method and urinary creatinine using the enzymatic colorimetric method. All analyses were done using Cobas 8000 Roche devices. For serum creatinine CV was < 2%, for serum cystatin C CV was < 3%, for urinary albumin CV was < 5% and for urinary creatinine CV was < 2%.

### Calibration of laboratory analyses

A calibration study of laboratory analyses on blood samples between the studies was conducted [20]. Regression equations derived from this study (Calibration plots Supplementary Fig. 1a and b) were used to correct for differences in methodology between the laboratory by adjusting the KYH serum creatinine measures by − 29.42 + 1.21*(original KYH measurement in μmol/L) and KYH cystatin c measures by 0.06 + 0.98*(original KYH measure in mg/L).

### Outcome variables

Serum creatinine was used to calculate eGFR using the creatinine based CKD-EPI eGFR formula to estimate renal function [23]. Serum creatinine alone was used as the primary measure of eGFR as this was available for the full subset of participants at Tromsø7.

A composite outcome labelled CKD was defined as reduced eGFR (< 60 ml/min/1.73 m2) and/or elevated albuminuria defined as ≥30 mg/g albumin/creatinine in urine. Repeat measures were not available in this study therefore the CKD outcome was based on single measures of eGFR and urinary albumin and creatine.

Differences in prevalence of reduced eGFR using serum creatinine were compared with those from using combined creatine and cystatin C in sensitivity analyses defined using the CKD-EPI combined creatinine-cystatin C equation [23].

### Risk factors

Medication classes were defined using the Anatomical Therapeutic Chemical (ATC) classification system [24]. For antihypertensive medication use, we combined answering “yes” to the questions “Do you use blood pressure lowering drugs and information from a self-reported list of brand names of regularly used medication as antihypertensives (ATC codes C02, C03, C07, C08 and C09). Hypertension was defined as ≥140 mmHg systolic and/or ≥90 mmHg diastolic at examination (mean of 2^nd^ and 3^rd^ measurement) or use of antihypertensives (ATC codes C02, C03, C07, C08 or C09 and/or yes to the question “Do you use of blood pressure lowering drugs?”). Hypertension was classified as a) uncontrolled untreated hypertension (raised blood pressure at examination/no use of antihypertensives); b) treated uncontrolled hypertension (raised blood pressure at examination/use of antihypertensives); c) controlled and treated (raised blood pressure at examination /use of antihypertensives) and d) normotensive (no raised blood pressure at examination/no use of antihypertensives). Diabetes was defined as HbA1c ≥ 6.5% [20] and/or self-report of diagnosis and/or self-reported use of diabetes medication (ATC codes A10B, A10A).

Information on education (lower, middle and higher coded based on education system in each country) and smoking status was collected from questionnaires. Education was defined as lower (incomplete secondary and vocational no secondary), middle (complete secondary, vocational and secondary, specialised secondary) and higher (incomplete higher, higher) education for KYH and lower (primary) middle (upper secondary) and higher (university/university college) for Tromsø7. Body mass index (BMI) was calculated from measured weight and height (kilograms/meter^2^ [kg/m^2^]). Abdominal obesity was defined as waist:hip ratio > 0.9. Due to differences in measurement protocols for waist circumference between the two studies (minimal waist for KYH, level of umbilicus for Tromsø7) waist circumference from Tromsø7 was converted to minimal waist using an established equation for the conversion [25]. All measurements were conducted by trained personnel.

### Statistical methods

#### Objective 1: comparison of prevalence of CKD between the two studies

The age- and sex- standardised prevalence of CKD, reduced eGFR and albuminuria were calculated with direct standardisation to the European 2013 standard population using 5 year age bands. Between-study differences in prevalence were investigated by fitting logistic regression models for each outcome with study as the exposure adjusted for age and sex. Models with and without interaction terms between study and a) age and b) sex were compared using likelihood ratio tests.

#### Objective 2: comparison of association with risk factors between studies

Risk factors for reduced eGFR and albuminuria were investigated for each study. Separate logistic regression analyses were run for reduced eGFR and albuminuria. In both models associations with age, sex, reduced eGFR (in the model for albuminuria) or albuminuria (in the model for reduced eGFR), education, smoking, BMI, waist: hip ratio, diabetes and hypertension were investigated. Models were adjusted 1) for age and sex (and city for KYH) and 2) mutually adjusted for all risk factors.

#### Objective 3: extent to which between-study differences in CKD were explained by established risk factors

The extent to which between-study differences in reduced eGFR and albuminuria were explained by established risk factors were investigated by fitting separate logistic regression models for each outcome with study as the main exposure and then adjusting systematically for risk factor variables one at a time and then all together. Here systolic and diastolic blood pressure and use of antihypertensive medication were adjusted for separately to disentangle the effects of the components of high blood pressure compared to possible effects from taking medications given medication use is related both to clinical indication and other wider health systems factors such as levels of awareness and treatment practices.

### Accounting for missing data

#### Objective 1

For KYH study missing data in the outcome was investigated by calculating inverse probability weights from age, age squared and sex taken from the sampling frame according to participation in the first stage separately for each city and then using a multiple imputation model [26] to impute missing outcomes using the factors associated with missingness at stages 1,2 and 3 shown in Fig. 1a. For Tromsø7 a multiple imputation model was used for outcomes which were measured only at the second visit (cystatin C, albuminuria). The predictors of missingness used were sex, age, age square, education (Fig. 1b) and reduced eGFR measured using serum creatinine. Individual level data on predictors of attendance at the first study visit were not available so this was not accounted for in the analysis. For both studies eGFR was used a priori in imputation models for albuminuria and CKD even if not associated with missingness in the outcomes.

Prevalence estimates for CKD overall (albuminuria and/or reduced eGFR) as well as prevalence estimates for reduced eGFR (using creatinine only and joint creatinine –cystatin C) and albuminuria separately were computed for both studies by sex and 10-year age bands. Analyses using survey weights were conducted using the survey-set suite in Stata, by reweighting the sample using the derived probability weights for each using robust variance estimators.

#### Objective 2

Chained multiple imputation [27] was used to take account of missingness in both the outcome and in the risk factors. Separate imputation models were used for the two outcomes and studies. For KYH data on reduced eGFR were imputed for all participants using both the risk factors in the model and predictors of missingness from stage 1 (Fig. 1a). For albuminuria this model would not converge therefore a simpler model using low eGFR and the measured risk factors (but not the additional predictors of missingness) from stage 1 was used to impute data for all participants who attended stage 2 of the study. For Tromsø7 models for both outcomes were imputed from measured risk factors for participants attending the basic examination of the study. The model for albuminuria additionally included low eGFR.

#### Objective 3

Chained multiple imputation (MI) models including both outcomes and all risk factors in the models were used for main analyses. These models were fitted separately for each study using dataset of all participants attending the health check for KYH and all participants attending the basic examination in Tromsø7 to create a consistent dataset for reduced eGFR and albuminuria. The MI datasets were then combined to conduct the analyses.

For all three objectives sensitivity analyses were conducted comparing findings with complete case analysis.

All analyses were conducted using Stata 16 [28].

The STROBE (Strengthening the reporting of observational studies in epidemiology) guidelines for the reporting of cross-sectional studies were followed in reporting the study findings.

## Results

### Prevalence of CKD

The prevalence of CKD among participants aged 40–69 years who provided blood and urine samples was 6.5% (95% CI 5.4, 7.7) in KYH and 4.6% (95% CI 4.0, 5.2) in Tromsø7 having standardised for sex and age. The age- and sex-adjusted prevalence was higher in the sample from Novosibirsk (8.0% (95% CI 5.6, 10.4) *n* = 548) than Arkhangelsk (6.0% (95% CI 4.8, 7.3) *n* = 1398) but with wide overlapping confidence intervals.

The age- and sex- adjusted odds ratio for CKD comparing KYH to Tromsø7 was 1.50 (95% CI 1.21, 1.86). There was no evidence for interaction in the between-study differences by age (likelihood ratio test for interaction *p* = 0.26) or sex (likelihood ratio test for interaction *p* = 0.22). However the absolute prevalence of CKD was substantially higher at older ages corresponding to larger prevalence differences between the studies in the oldest age group (60–69 years).

**Fig. 2:**
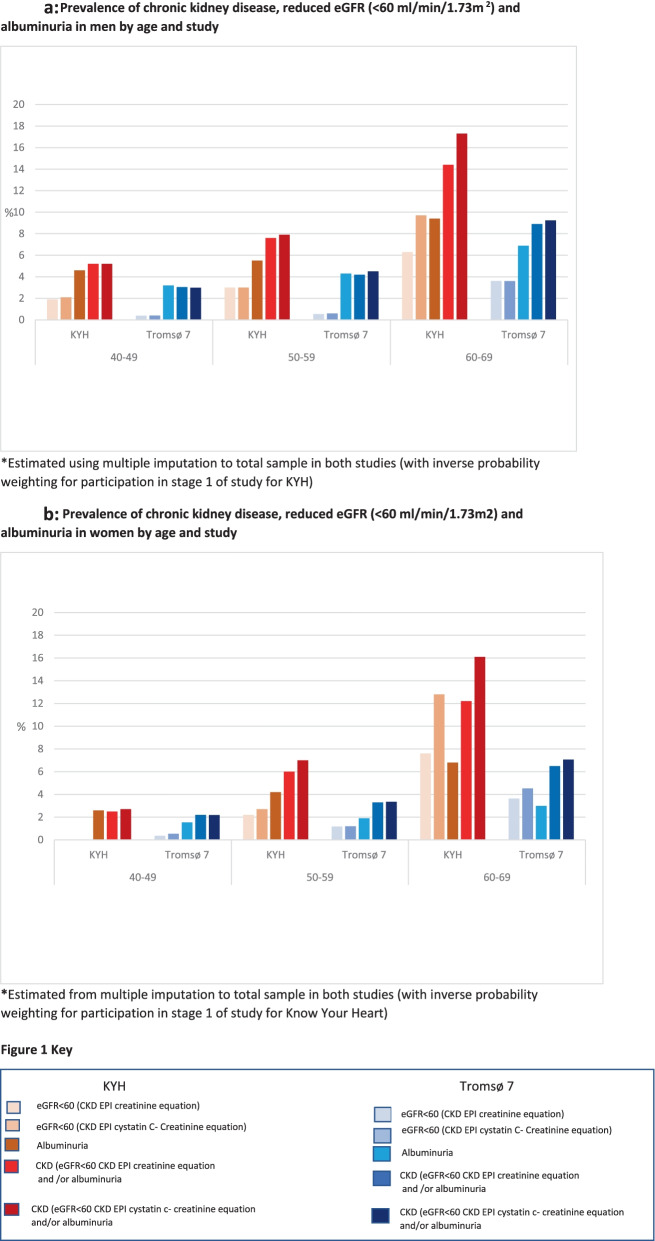
Prevalence of chronic kidney disease, reduced eGFR (< 60 ml/min/1.73m^2^) and albuminuria by age and study stratified by sex. **a** Prevalence of chronic kidney disease, reduced eGFR (< 60 ml/min/1.73m^2^) and albuminuria in men by age and study. **b** Prevalence of chronic kidney disease, reduced eGFR (< 60 ml/min/1.73m^2^) and albuminuria in women by age and study

The prevalence of CKD, reduced eGFR (creatinine only, and joint creatinine and cystatin C equation) and albuminuria in KYH and Tromsø7 by age and sex are shown in Fig. 2a and b. For all outcomes prevalence was higher in KYH than in Tromsø7 in both sexes for every age group. The exception was among women aged 40–49 years where there was no evidence for a difference between the two study populations.

### Associations with risk factors

The distributions of risk factors in the two studies by sex are shown in Table 1.

**Table 1:** Distribution of Risk factors for chronic kidney disease by study and sex^*^

		**Know Your Heart**						**Tromsø7**					
		**Men**		**Women**		**Total**		**Men**		**Women**		**Total**	
		**N**	**(%)**	**N**	**(%)**	**N**	**(%)**	**N**	**(%)**	**N**	**(%)**	**N**	**(%)**
All		1700	(100)	2346	(100)	4046	(100)	9261	(100)	8309	(100)	17,561	(100)
Age	40–49	470	(27.7)	690	(29.4)	1160	(28.7)	3043	(36.6)	3366	(36.4)	6409	(36.5)
	50–59	563	(33.1)	774	(33.0)	1337	(33.0)	2773	(33.4)	3232	(34.9)	6005	(34.2)
	60–69	667	(39.2)	882	(37.6)	1548	(38.3)	2493	(30.0)	2663	(28.8)	5156	(29.4)
City	Arkhangelsk	887	(52.2)	1232	(52.5)	2119	(52.4)	–		–		–	
	Novosibirsk	813	(47.8)	1114	(47.5)	1927	(47.6)	–		–		–	
Education	Low	147	(8.7)	134	(5.7)	281	(6.9)	1610	(19.6)	1680	(18.3)	3290	(18.9)
	Middle	878	(51.7)	1280	(54.6)	2158	(53.5)	2538	(30.9)	2386	(26.0)	4942	(28.3)
	Higher	675	(39.7)	932	(39.7)	1607	(39.7)	4079	(49.6)	5105	(55.7)	9184	(52.8)
	Missing							90		82		172	–
Smoking status	Never smoker	432	(25.5)	1595	(68.2)	2027	(50.3)	3636	(44.1)	3748	(40.7)	7384	(42.3)
	Ex-smoker	640	(37.8)	370	(15.8)	1010	(25.0)	3434	(41.6)	4022	(43.7)	7456	(42.8)
	Current smoker	622	(36.7)	375	(16.0)	997	(24.7)	1179	(14.3)	1422	(15.5)	2601	(14.9)
	Missing	6		6		12		60		69		129	
Body mass index	< 18.5	18	(1.1)	27	(1.2)	45	(1.1)	14	(0.2)	74	(0.8)	88	(0.5)
	18.5–24.9	467	(27.5)	653	(27.9)	1120	(27.8)	1930	(23.3)	3777	(40.9)	5707	(32.6)
	25.0–29.9	754	(44.5)	762	(32.6)	1516	(37.6)	4213	(50.8)	3353	(36.3)	7566	(43.2)
	30.0–34.9	349	(20.6)	550	(23.5)	899	(22.3)	1678	(20.2)	1432	(15.5)	3110	(17.7)
	> 35	108	(6.4)	346	(14.8)	454	(11.3)	456	(5.5)	602	(6.5)	1058	(6.0)
	Missing					12		18		23		41	
Waist: Hip ratio	< 0.9	402	(23.7)	545	(76.8)	2202	(54.5)	2297	(27.8)	93.9	(93.9)	10,988	(62.8)
	> 0.9	1297	(76.3)	1800	(23.2)	1842	(45.6)	5980	(72.3)	537	(5.8)	6517	(37.2)
	Missing	1		1		2		33		32		65	
Mean SBP (SD)		137.9	(19.6)	128.9	(19.7)	132.7	(20.1)	132.5		127.0	(20.8)	129.6	(19.8)
Mean DBP (SD)		86.2	(11.2)	80.8	(11.0)	83.1	(11.4)	78.3	(9.8)	72.8	(9.7)	75.4	(10.1)
Hypertension (measured blood pressure/use of antihypertensives)	Normotensive	496	(32.1)	853	(40.5)	1349	(36.9)	4848	(58.5)	6493	(70.3)	11,341	(64.7)
	Hypertensive (treated and controlled)	268	(17.4)	593	(28.1)	861	(23.6)	981	(11.8)	939	(10.2)	1920	(11.0)
	Hypertensive (untreated)	384	(24.9)	198	(9.4)	582	(15.9)	1701	(20.5)	1219	(13.2)	2920	(16.7)
	Hypertensive (treated, uncontrolled)	396	(25.7)	465	(22.1)	861	(23.6)	762	(9.2)	582	(6.3)	1344	(7.7)
	Missing	156		237		393		17		28		45	
Diabetes (self-report or use of medication or HbA1c ≥ 6.5%)	No	1461	(87.2)	1961	(85.0)	3422	(85.9)	7719	(93.6)	8776	(95.5)	16,495	(94.6)
	Yes	215	(12.8)	347	(15.0)	562	(14.1)	526	(6.4)	410	(4.5)	936	(5.4)
	Missing	24		38		62		64		75		139	
Total		1700	(42.0)	2346	(100)	4046	(100)	8309	(100)	9261	(100)	17,570	(100)

^*^Study population men and women aged 40–69 with data on serum creatinine

The associations of various risk factors with reduced eGFR in Russia and Norway are shown in Table 2.

**Table 2:** Association of risk factors for reduced eGFR estimated from serum creatinine by study^#^

		**Know Your Heart (MI dataset** ***N*** **= 4607)**				**Tromsø7 (MI dataset** ***N*** **= 17,646)**			
		**n/Total N**	**Reduced eGFR < 60 ml/min/1.73 m2** ^**§**^ **(%)**	Age sex and city adjusted OR^*#^	Mutually adjusted OR^**#^ **(95% CI)**	**n/Total N**	**Reduced eGFR < 60 ml/min/1.73 m2 (%)**^**#**^	Age and sex adjusted OR^*,#^ **(Multiple imputation)**	Mutually adjusted OR^**, #^ **(95% CI)**
Total sample		145/4046	3.58			264/17,570	1.50		
Age	40–49	7/1160	0.10	1.00 (ref)	1.00 (ref)	24/6409	0.38	1.00 (ref)	1.00 (ref)
	50–59	32/1337	2.60	3.85 (1.69, 8.75)	2.98 (1.30,6.87)	53/6005	0.88	2.36 (1.45, 3.82)	1.91(1.17, 3.12)
	60–70	106/1549	6.89	11.24 (5.21, 24.23)	6.84 (3.07, 15.24)	187/5156	3.63	10.00 (6.53, 15.32)	5.88 (3.73, 9.26)
	Test for trend			*P* < 0.001	*P* < 0.001			*P* < 0.001	*P* < 0.001
Sex	Men	81/2346	3.86	1.00 (ref)	1.00 (ref)	147/9261	1.59	1.00 (ref)	1.00 (ref)
	Women	64/1700	3.16	0.94 (0.67, 1.31)	0.84 (0.53, 1.33)	117/8309	1.41	1.16 (0.91, 1.48)	1.84 (1.25, 2.71)
Education (missing imputed Tromsø7 = 174)	Low	18/281	5.64	1.43 (0.83, 2.45)	1.45 (0.84, 2.50)	82/3290	2.52	1.32 (0.95, 1.85)	1.29 (0.92, 1.80)
	Middle	77/2158	3.53	1.00 (ref)	1.00 (ref)	66/4924	1.36	1.00 (ref)	1.00 (ref)
	Higher	50/1607	3.12	0.98 (0.68, 1.41)	0.98 (0.67, 1.43)	110/9184	1.21	1.01 (0.74, 1.37)	1.10 (0.80, 1.51)
	Test for trend			*P* = 0.33	*P* = 0.31			*P* = 0.09	*P* = 0.34
Smoking status (missing imputed KYH = 451; Tromsø7 = 130)	Never smoker	83/2027	3.96	1.00 (ref)	1.00 (ref)	97/7384	1.33	1.00 (ref)	1.00 (ref)
	Ex-smoker	27/1010	3.15	0.64 (0.39, 1.04)	0.60 (0.37, 0.99)	132/7456	1.79	1.03 (0.78, 1.34)	1.06 (0.72,1.24)
	Current smoker	34/997	3.24	0.98 (0.62, 1.55)	1.05 (0.65,1.67)	30/2601	1.17	0.73 (0.48,1.11)	0.71 (0.46, 1.10)
Body mass index (missing imputed KYH = 454; Tromsø7 = 43)	< 18.5	0/45	0.00	1.00 (ref)	1.00 (ref)	2/88	2.27	2.03 (0.48, 8.58)	2.38 (0.56, 10.21)
	18.5–24.9	24/1120	2.19			56/5707	0.98	1.00 (ref)	1.00 (ref)
	25.0–29.9	50/1516	3.07	1.26 (0.76,2.07)	1.13 (0.68, 1.89)	118/7566	1.56	1.57 (1.14,2.17)	1.31 (0.94, 1.82)
	30.0–34.9	45/899	4.79	1.80 (1.08, 3.01)	1.39 (0.81, 2.39)	72/3110	2.32	2.41 (1.69,3.44)	1.62 (1.11, 2.35)
	> 35	25/453	5.74	2.01 (1.11, 3.63)	1.36 (0.72, 2.57)	13/1057	1.22	1.44 (0.78,2.64)	0.76 (0.40,1.42)
	Test for trend			*P* = 0.005	*P* = 0.23			*P* < 0.001	*P* = 0.49
Waist: Hip ratio (missing imputed KYH = 444; Tromsø7 = 74)	< 0.9	55/2202	2.45	1.00 (ref)	1.00 (ref)	136/10988	1.25	1.00(ref)	1.00(ref)
	> 0.9	89/1842	4.58	1.75 (1.18, 2.59)	1.48 (0.98, 2.24)	125/6517	1.94	2.13 (1.47, 3.10)	1.60 (1.09, 2.35)
Albuminuria^***^ (missing imputed KYH = 2817) Tromsø7 = 12,126)	No	44/1677	2.88	1.00 (ref)	1.00 (ref)	111/5284	1.26	1.00(ref)	1.00 (ref)
	Yes	12/82	16.09	4.89 (2.60, 9.20)	4.40 (2.26, 8.57)	25/217	8.45	6.12 (3.78, 9.93)	5.14 (3.05, 8.66)
Hypertension (measured blood pressure/use of antihypertensives) Missing imputed KYH = 788; Tromsø7 = 53)	Normotensive	19/1349	1.41	1.00 (ref)	1.00 (ref)	85/11,341	0.75	1.00 (ref)	1.00 (ref)
	Hypertensive (treated and controlled)	48/861	5.85	2.59 (1.48, 4.54)	2.35 (1.31, 4.21)	88/1920	4.59	3.90 (2.84, 5.35)	3.45 (2.48, 4.80)
	Hypertensive (untreated)	10/582	2.11	0.90 (0.41,1.95)	0.83 (0.38, 1.81)	30/2920	1.03	0.99 (0.65, 1.51)	0.92 (0.60, 1.42)
	Hypertensive (treated, uncontrolled)	52/861	6.44	2.52 (1.45, 4.38)	2.24 (1.27, 3.97)	60/1344	4.47	3.46 (2.43,4.92)	3.01 (2.09,4.35)
Diabetes (self-report or use of medication or HbA1c ≥ 6.5%) (missing imputed KYH =510; Tromsø7 202)	No	107/3422	3.06	1.00 (ref)	1.00 (ref)	222/16442	1.35	1.00 (ref)	1.00 (ref)
	Yes	38/562	6.57	1.54 (1.04, 2.27)	1.10 (0.72, 1.68)	39/989	4.10	2.30 (1.61, 3.28)	1.47 (1.01, 2.14)

^*^Adjusted for age, sex, city (If Russia) ^**^Adjusted for age, sex, city (Know Your Heart only), education, body mass index, waist hip ratio (body mass index and waist hip ratio not mutually adjusted for each other), smoking status, hypertension, diabetes

^***^Imputed in KYH using simpler model with minimal covariates from baseline (age, age squared, sex and education) for all health check attendees *N* = 4122

^§^Estimated from chained multiple imputation model with survey weights for non-response by age and sex

^#^Estimated from chained multiple imputation model

The pattern of association after adjusting for age and sex was similar in both studies with large increases in prevalence with age and positive associations with albuminuria, hypertension, diabetes, high BMI and waist to hip ratio in Norway. The increased odds of reduced eGFR by hypertension were found in those who were treated regardless of whether hypertension was controlled according to measured blood pressure at the time of the survey but not in those who were hypertensive but not treated. There was no evidence for an association with current smoking and reduced eGFR in either study.

The associations with albuminuria in KYH and Tromsø7 are shown in Table 3. Older age, hypertension, diabetes and BMI > 35 kg/m^2^ in both studies and current smoking in Tromsø7 were associated with higher odds of albuminuria. There was no sex difference in KYH but Tromsø7 women had lower odds of albuminuria than men. In both studies the strongest association found after mutual adjustment for other risk factors was with uncontrolled treated hypertension status. It is worth noting that there was much higher prevalence of uncontrolled treated hypertension in KYH compared with Tromsø7 (Table 1).

**Table 3:** Association with risk factors for elevated albuminuria (≥30 mg/g albumin/creatinine) by study^c^

		**Know Your Heart (MI dataset** ***N*** **= 4122)**				**Tromsø7 (MI dataset** ***N*** **= 17,646)**			
		**n/Total n**	**(%)**^**c**^	**Age, sex and city adjusted OR**^**ac**^**(95% CI)**	**Mutually adjusted OR**^**bc**^ **(95% CI)**	**n/Total n**	**(%)**^**c**^	**Age and sex adjusted OR**^**ac**^ **(95% CI)**	**Mutually adjusted OR**^**bc**^ **(95% CI)**
Total		84/1790	5.76			218/5520	3.41		
Age	40–49	15/491	3.86	1.00 (ref)	1.00 (ref)	25/1029	2.26	1.00 (ref)	1.00 (ref)
	50–59	21/610	4.69	1.22 (0.63, 2.36)	0.90 (0.45, 1.79)	39/1300	3.28	1.50 (1.00, 2.26)	1.22 (0.80, 1.86)
	60–70	48/689	8.07	2.14 (1.19, 3.84)	1.23 (0.63, 2.37)	154/3191	5.01	2.32 (1.50, 3.58)	1.53 (0.97, 2.42)
	Test for trend			*P* = 0.005	*P* = 0.42			*P* < 0.001	*P* = 0.06
Sex	Men	43/749	6.73	1.00 (ref)	1.00 (ref)	141/2506	4.87	1.00 (ref)	1.00 (ref)
	Women	41/1041	5.05	0.74 (0.49, 1.14)	0.72 (0.37, 1.39)	77/3014	2.11	0.42 (0.32, 0.56)	0.48 (0.31, 0.72)
Education (missing imputed T7 = 174)	Low	11/120	9.09	1.38 (0.73, 2.60)	1.41 (0.72, 2.73)	66/1292	4.97	1.16 (0.82, 1.63)	1.04 (0.73, 1.49)
	Middle	41/923	5.88	1.00 (ref)	1.00 (ref)	68/1610	3.93	1.00 (ref)	1.00 (ref)
	Higher	32/747	5.01	0.87 (0.57, 1.34)	1.02 (0.66, 1.59)	79/2554	2.58	0.71 (0.52, 0.97)	0.88 (0.64, 1.22)
	Test for trend			*P* = 0.27	*P* = 0.62			*P* = 0.002	*P* = 0.30
Smoking status (missing imputed Russia = 12; T7 = 130)	Never smoker	40/957	5.27	1.00 (ref)	1.00 (ref)	66/2071	2.71	1.00 (ref)	1.00 (ref)
	Ex-smoker	21/468	5.38	0.95 (0.51, 1.77)	0.90 (0.47, 1.75)	95/2615	3.12	1.07 (0.80, 1.43)	0.98 (0.72, 1.32)
	Current smoker	22/363	7.13	1.39 (0.80, 2.41)	1.58 (0.89, 2.80)	55/792	6.23	2.31 (1.54, 3.56)	2.19 (1.45, 3.30)
Body mass index (missing imputed Russia = 16, T7 = 43)	< 18.5	0/14	0.0	1.00(ref)	1.00 (ref)	5/34	14.30	7.98 (3.02,21.07)	6.58 (2.39, 18.12)
	18.5–24.9	17/513	4.07			48/1752	2.38	1.00 (ref)	1.00 (ref)
	25.0–29.9	5/674	4.37	1.01 (0.53, 1.91)	0.82 (0.42, 1.59)	84/2445	2.98	1.02 (0.73, 1.43)	0.92 (0.64, 1.31)
	30.0–34.9	20/406	6.44	1.53 (0.83, 2.80)	1.00 (0.52, 5.23)	50/969	4.49	1.50 (0.98, 2.28)	1.19 (0.79, 1.80)
	> 35	20/178	12.91	3.76 (1.99, 7.09)	2.24 (1.11, 4.52)	38/306	7.77	3.35 (2.00, 5.61)	2.09 (1.27, 3.43)
	Test for trend			*P* < 0.001	*P* = 0.03			*P* < 0.001	*P* = 0.04
Waist: Hip ratio (missing imputed Russia = 5; Norway = 74)	< 0.9	33/964	4.36	1.00 (ref)	1.00 (ref)	83/3361	2.20	1.00 (ref)	1.00 (ref)
	> 0.9	50/825	7.42	1.53 (0.90,2.63)	1.04 (0.59, 1.82)	132/2143	5.46	1.70 (1.15, 2.51)	1.19 (0.81, 1.75)
Reduced eGFR based on serum creatinine (missing imputed KYH = 75 T7 = 76)	No	69/1707	5.07	1.00 (ref)	1.00 (ref)	192/5365	3.18	1.00 (ref)	1.00 (ref)
	Yes	14/73	24.26	4.88 (2.60, 9.16)	4.32 (2.20, 8.46)	25/136	19.13	6.09 (3.76, 9.89)	5.13 (3.04, 8.68)
Hypertension (measured blood pressure/use of antihypertensives) (missing imputed Russia = 398, T7 = 53)	Normotensive	10/607	2.31	1.00 (ref)	1.00 (ref)	66/3030	2.01	1.00 (ref)	1.00 (ref)
	Hypertensive (treated and controlled)	16/421	4.62	1.95 (0.85, 4.52)	1.43 (0.59, 3.49)	59/816	6.84	2.98 (2.08, 4.28)	2.33 (1.56, 3.48)
	Hypertensive (untreated)	9/242	5.44	2.21 (0.90, 5.41)	1.92 (0.76, 4.80)	47/1063	4.62	1.96 (1.35, 2.84)	1.77 (1.20, 2.60)
	Hypertensive (treated, uncontrolled)	39/376	12.51	5.60 (2.56, 12.28)	4.30 (1.92, 9.62)	46/599	7.74	3.22 (2.16, 4.82)	2.59 (1.71, 3.94)
Diabetes (self-report or use of medication or HbA1c ≥ 6.5%) (missing imputed Russia = 130 T7 = 200)	No	56/1501	4.67	1.00 (ref)	1.00 (ref)	173/5105	2.99	1.00 (ref)	1.00 (ref)
	Yes	25/249	12.29	2.54 (1.60, 4.04)	1.91 (1.13, 3.22)	43/364	10.94	3.33 (2.33, 4.75)	2.17 (1.47, 3.19)

^a^Adjusted for age, sex, city (KYH only) ^b^ Adjusted for age, sex, city (Know Your Heart only), education, body mass index, waist hip ratio (body mass index and waist hip ratio not mutually adjusted for each other), smoking status, hypertension, diabetes

^c^Estimated from chained multiple imputation model

### Extent to which between-study differences in the prevalence of reduced eGFR and albuminuria are explained by differences in known risk factors in the study populations

The associations between reduced eGFR and albuminuria adjusting for known risk factorsare shown in Table 4.

**Table 4:** Association between Study (Know Your Heart /Tromsø7^∞^) and reduced eGFR and albuminuria adjusting for known risk factors^§^

	**Reduced eGFR < 60 ml/min/1.73m**^**2**^ **(%)**		**Elevated albuminuria ≥30 mg/g albumin/creatinine**	
	**OR (95% CI)**	***P*** **value**	**OR (95% CI)**	***P*** **value**
+ Age + Sex	2.06 (1.67, 2.54)	< 0.001	1.54 (1.16, 2.03)	0.003
+ Age + sex+ education	2.19 (1.76, 2.73)	< 0.001	1.54 (1.16, 2.03)	0.003
+ Age + sex + smoking status	2.07 (1.67, 2.56)	< 0.001	1.47 (1.11, 1.94)	0.007
+ Age + sex + body mass index + waist: hip ratio	1.83 (1.47, 2.27)	< 0.001	1.37 (1.03, 1.82)	0.03
+ Age + sex + mean systolic blood pressure	1.95 (1.57, 2.42)	< 0.001	1.30 (0.98, 1.73)	0.07
+ Age + sex + mean diastolic blood pressure	1.99 (1.58, 2.50)	< 0.001	1.18 (0.96, 1.59)	0.29
+ Age + sex + mean systolic blood pressure + mean diastolic blood pressure	2.08 (1.65, 2.63)	< 0.001	1.29 (0.94, 1.74)	0.12
+ Age + sex + antihypertensive medication	1.45 (1.16, 1.80)	0.001	1.21 (0.91, 1.60)	0.18
+ Age + sex + diabetes	1.89 (1.52, 2.33)	< 0.001	1.35 (1.01, 1.79)	0.04
+ Age and sex + smoking+ body mass index + waist: hip ratio+ + mean diastolic blood pressure + diabetes	1.70 (1.33, 2.17)	< 0.001	0.91 (0.66, 1.25)	0.56
+ Age and sex + smoking+ body mass index + waist: hip ratio + mean systolic blood pressure + mean diastolic blood pressure + diabetes	1.83 (1.43, 2.33)	< 0.001	1.05 (0.77, 1.43)	0.76
+ Age and sex + smoking+ body mass index + waist: hip ratio + mean systolic blood pressure + mean diastolic blood pressure + diabetes + antihypertensive medication	1.42 (1.11, 1.82)	0.006	0.92 (0.68, 1.25)	0.59

^∞^Reference population is Tromsø7

^§^ Estimated from chained multiple imputation

*Study population all participants attending health check stage (Know Your Heart) and Basic Examination (Tromsø7)

Adjustment for all risk factors except education and measured mean systolic and diastolic blood pressure attenuated the between-study difference in reduced eGFR. The largest difference in odds ratio was seen on adjustment for use of antihypertensive medication. Adjustment for all risk factors together attenuated the odds ratio substantially but did not completely explain the between-study difference.

For albuminuria the between-study difference was smaller than for reduced eGFR. The individual risk factors which reduced the between study difference the most were mean diastolic blood pressure followed by use of antihypertensive medication. In contrast with reduced eGFR adjusting for all risk factors reduced the between study difference to the null.

Findings for all three objectives were substantively similar for the complete case analysis (Supplementary Fig. 2a and b; Supplementary Tables 1, 2, 3).

Distribution of serum creatinine and urinary albumin to creatinine ratio by study are shown in Supplementary Figs. 3 and 4.

## Discussion

In this study we compared the prevalence of CKD in adults aged 40–69 years participating in population-based studies in Russia and Norway. The prevalence of reduced eGFR, albuminuria and CKD as a composite outcome (reduced eGFR and/or albuminuria) were all substantially higher in the Russian sample than the Norwegian sample.

The association between established risk factors and reduced eGFR and albuminuria were similar in the two study populations. This is reassuring, as it means that there is a clear biological impact of the established risk factors which does not appear to depend on geography. However, the distribution of risk factors in the two studies was different with a higher prevalence of hypertension, especially uncontrolled treated hypertension, diabetes, obesity (in women) and smoking (among men) in the Russian sample with associated sex differences in CKD prevalence that were different between Norway and Russia.

While the higher prevalence of albuminuria in the Russian sample appeared to be explained by differences in established risk factors between the study populations, the difference in prevalence of reduced eGFR was only partially explained. For both reduced eGFR and albuminuria differences in the use of antihypertensive medication was a strong explanatory factor for the between-study difference in prevalence, while mean diastolic blood pressure was a strong explanatory factor for the differences in albuminuria. There is still ongoing discussion as to whether high blood pressure is the first sign of kidney damage as opposed to being a risk factor for kidney disease. Genetic data suggest that reduced eGFR causes high blood pressure but not vice versa [29], whilst albuminuria and blood pressure have a bidirectional association [30]. Hence, any interpretation of these prevalence data of CKD relative to blood pressure and to which extent the CKD prevalence would be modifiable by intervening on blood pressure earlier is very challenging. If one believes that high blood pressure is the earliest manifestation of kidney damage which if untreated is followed by eGFR decline (accelerated by the presence of albuminuria), then our findings are very worrying indeed, as then it appears that there is a much higher prevalence of kidney damage in Russia. By better addressing the extent of underlying factors that drive kidney damage (such as for example longstanding overweight), some of the long term impacts of high blood pressure and CKD could be prevented. In line with this hypothesis, we find that despite blood pressure being treated more often in Russia compared to Norway there is a challenge to control high blood pressure [31], as is typical for people with underlying kidney disease. The interpretation of causal relationships between CKD and blood pressure are beyond the scope of this paper but our findings are important for highlighting the potential high burden of CKD within the Russian population which calls for further investigation. It is a limitation of this study is that the data are cross-sectional and we were unable to capture differences in exposure to risk factors over time. Given limitations due to the cross-sectional nature of the data as regards any causal inference a formal estimation of the population attributable risk fraction following approaches from case-control [32] or cohort studies [33] was not conducted.

We also consider that the associations seen here are likely to be an underestimate of the association between kidney function markers and blood pressure, because blood pressure shows high within person variability and measurements at one point in time. Additionally this may be raised in response to anxiety over the measurement (“white coat” hypertension) may well not capture true differences in having lived with raised blood pressure over a longer period of time. Differences in measured blood pressure at the time of the health check did not explain between-study differences in reduced eGFR although mean DBP was important in explaining the between-study differences in albuminuria. We hypothesise that our findings with respect to use of antihypertensive medication may be a reflection that this is a better marker of chronic raised blood pressure than BP measurements taken at one point within a clinical setting.

There is substantial variation in the prevalence of CKD throughout Europe. Here we found an age-and sex-standardised prevalence of 6.4% in the Russian sample and 4.6% in the Norwegian sample. Our findings are in line with a relatively low prevalence of CKD in Norway (3.3% in the HUNT study from central Norway) compared to other European counties in a multi-site comparison of adults aged 45–64 where prevalence ranged from 3 to 19% [3] while the estimates for Russia are also in the lower range for this study. A previous population-based study from Novosibirsk also indicated low prevalence of reduced eGFR (0.3% of the study sample) [19], however given the study was among young adults (aged 25–45 years) it is hard to draw conclusions from this as to prevalence at older ages. The finding that CKD prevalence is not high in Russia compared to International studies is perhaps surprising given the very high burden of CVD mortality in Russia. It is not possible to know from our data whether this is due to selection bias in our study population or reflects the true prevalence, perhaps influenced by differential survival due to high levels of premature CVD mortality.

A strength of our study was the opportunity to compare harmonised data collected in the same time frame using both urinary and blood testing to classify CKD status as per the 2012 KDIGO guidelines [34]. However, we must also consider the potential for selection bias as a limitation given levels of non-response at each stage of the study including initial participation in the study (30% of initial addresses issued for KYH, 65% Tromsø7) and the possibility that our study populations were not representative of the underlying target populations they were selected to represent. Urine samples were provided in both KYH and Tromsø7 by a subset of participants only. Here we investigated the impact of missing data at different stages of the studies using a combination of multiple imputation and inverse probability weighting and found our results were robust to different methods of handling missingness. However, there are some limitations to this as we did not have data beyond age and sex for those who did not take part at any stage of the KYH study or Tromsø7. Additionally, we cannot generalise results to the whole of Russia and Norway given data were from two cities in Russia and one (mainly urban) municipality in Norway. There was some evidence for variation between the two Russian study populations in the prevalence of CKD and a National level study is needed to estimate the burden of CKD across Russia. Furthermore, as this was a cross-sectional study therefore the temporal relationship between CKD and the risk factors considered could not be established and only one measurement of eGFR and urine/albumin creatinine ratio was available.

Finally, there are potential limitations with regards potential for measurement error within the study. Although data collection was harmonised where possible including a laboratory calibration study for serum creatinine and cystatin C, the equivalent was not done for urine samples and there were some differences between the studies in measurement protocols, notable analysis of fresh urine for Tromsø7 compared to frozen urine samples for KYH. However, although there was some variation in the effect size for the difference between KYH and Tromsø7 depending on the measure of kidney damage used, the substantive pattern of results was consistent across measures.

## Conclusions

In conclusion, we found clear evidence for a higher burden of CKD within a sample of the Russian general population compared to a Norwegian sample. Differences in established risk factors between the study populations partly explained this. Hypertension defined through medication use was a major factor associated with the higher prevalence of CKD in the Russian study population.

## Supplementary Information

**Additional file 1.**

**Additional file 2.**

**Additional file 3.**

**Additional file 4.**

**Additional file 5.**

## Data Availability

The data that support the findings of this study are available from Know Your Heart [International Project on Cardiovascular Disease in Russia | Know your heart] and The Tromsø Study [The Tromsø Study | UiT], but restrictions apply to the availability of these data, which were used under license for the current study, and so are not publicly available. Data from the Know Your Heart Study are available from the authors upon reasonable request with permission of Know Your Heart Study. For The Tromsø Study, data are available upon reasonable request, subject to permission from The Tromsø Study which requires scientific and ethical approval of a protocol.

## Abbreviations

ATC: Anatomic Therapeutic Chemical
BMI: Body mass index
CI: Confidence Interval
CKD: Chronic Kidney Disease
CVD: Cardiovascular Disease
CV: Coefficient of Variance
DBP: Diastolic blood pressure
eGFR: Estimated Glomerular Filtration Rate
KDIGO: Kidney Disease Improving Global Outcomes
KYH: Know Your Heart
MI: Multiple imputation
SBP: Systolic blood pressure
SD: Standard deviation
STROBE: Strengthening the reporting of observational studies in epidemiology
