# Supplementary material for: Comparing prevalence of chronic kidney disease and its risk factors between population-based surveys in Russia and Norway
Source: BMC Nephrol. 2022 Apr 14;23:145. doi: 10.1186/s12882-022-02738-2 (PMC9008943; doi:10.1186/s12882-022-02738-2)
Supplement: Supplementary file 1 — Additional file 1. [file 12882_2022_2738_MOESM1_ESM.pdf]

Supplementary Figure 1a: Scatter and differential (Bland-Altman) plots of Lytech Laboratory (Moscow) versus UNN Laboratory (Tromsø) assayed serum creatinine the KYH recalibration subsample*

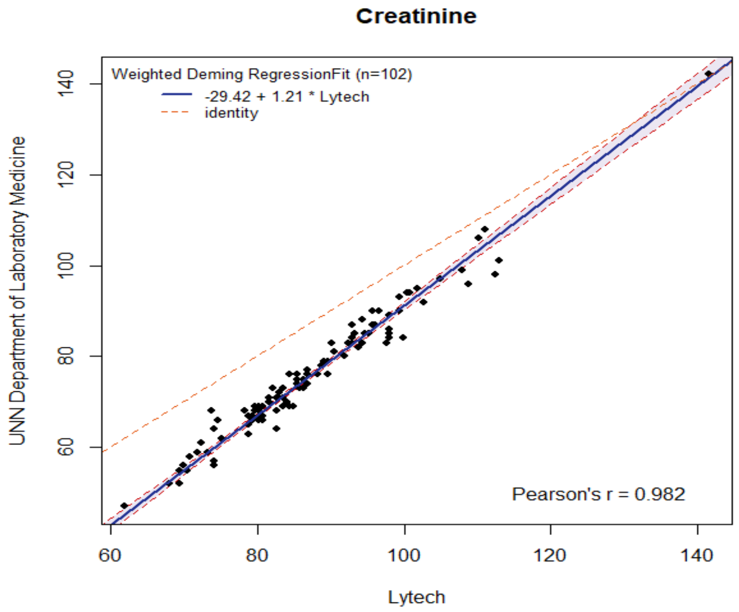

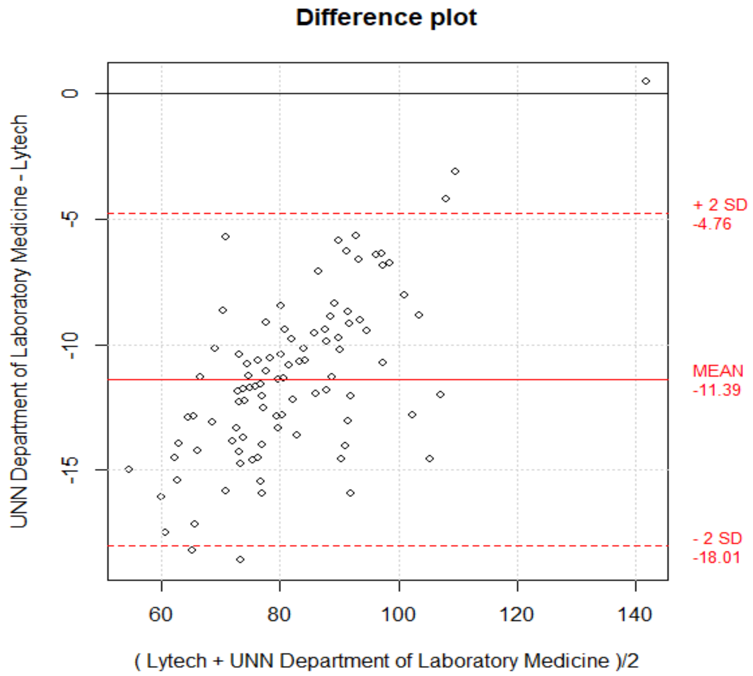

* The resulting equation -29.42 + 1.21*Creatinine was used for conversion of KYH values. For the detailed description of the calibration study see Iakunchykova O, Averina M, Wilsgaard T, *et al* Why does Russia have such high cardiovascular mortality rates? Comparisons of blood-based biomarkers with Norway implicate non-ischaemic cardiac damage *J Epidemiol Community Health* 2020;**74:**698-704.

Supplemental Figure 1b. Scatter and differential (Bland-Altman) plots of Lytech laboratory (Moscow) versus UNN laboratory (Tromsø) assayed cystatin C in the KYH recalibration subsample*

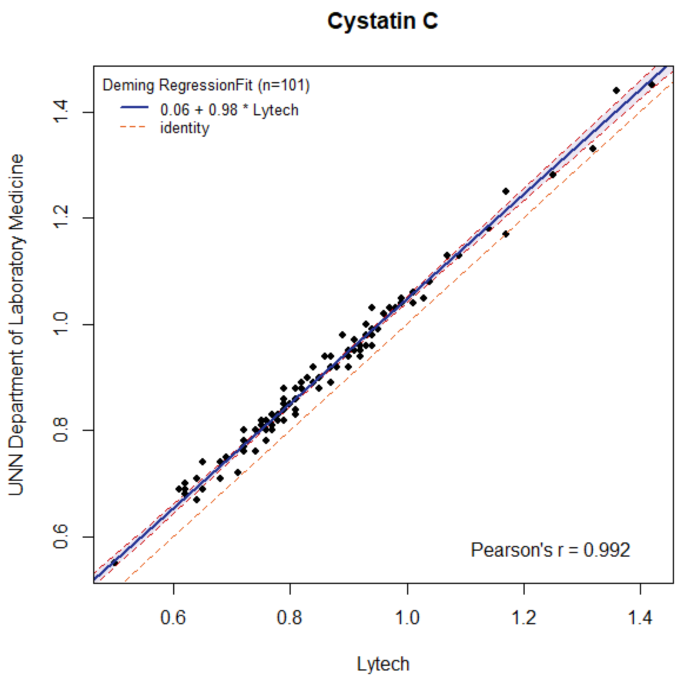

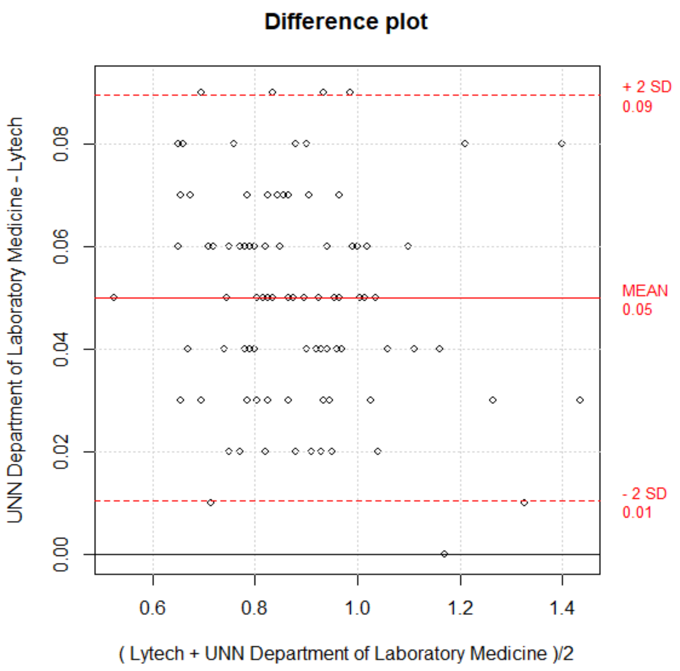

*The resulting equation 0.06 + 0.98*Cystatin C was used for conversion of KYH values. For the detailed description of the calibration study see: Iakunchykova O, Averina M, Wilsgaard T, *et al* Why does Russia have such high cardiovascular mortality rates? Comparisons of blood-based biomarkers with Norway implicate non-ischaemic cardiac damage *J Epidemiol Community Health* 2020;**74:**698-704.
