# Supplementary material for: Comparing prevalence of chronic kidney disease and its risk factors between population-based surveys in Russia and Norway
Source: BMC Nephrol. 2022 Apr 14;23:145. doi: 10.1186/s12882-022-02738-2 (PMC9008943; doi:10.1186/s12882-022-02738-2)
Supplement: Supplementary file 2 — Additional file 2. [file 12882_2022_2738_MOESM2_ESM.pdf]

Supplementary Figure 2a: Prevalence of chronic kidney disease, reduced eGFR (<60 ml/min/1.73m^2) and albuminuria in men by age and study (complete case analysis)

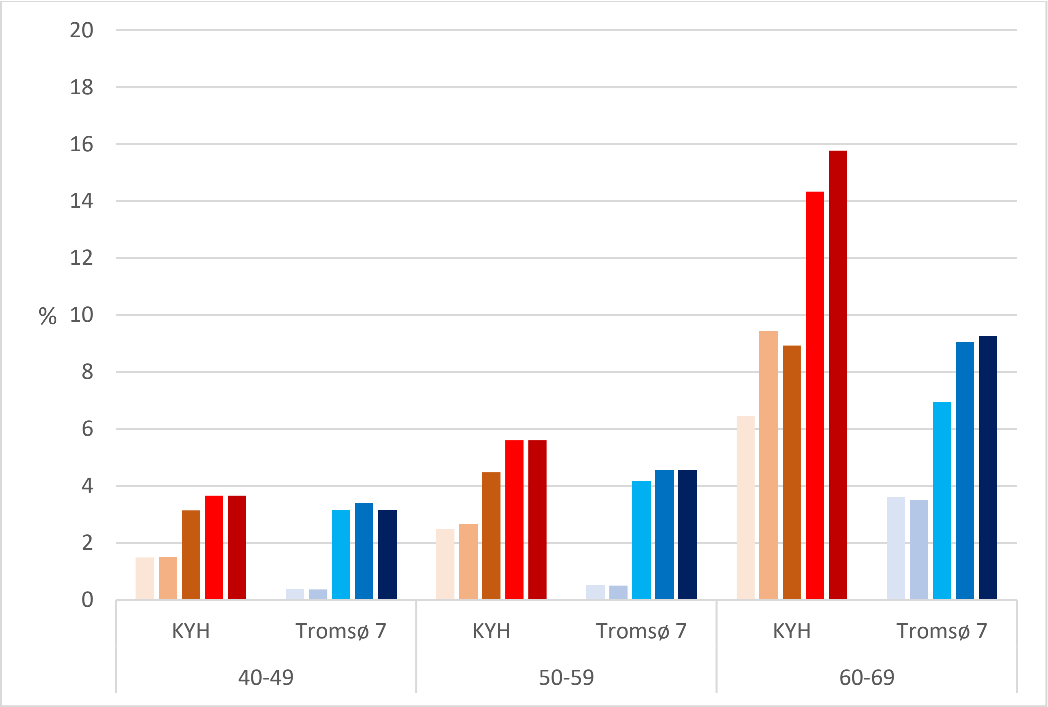

Supplementary Supplementary Figure 2b: Prevalence of chronic kidney disease, reduced eGFR (<60 ml/min/1.73m^2) and albuminuria in women by age and study (complete case analysis)

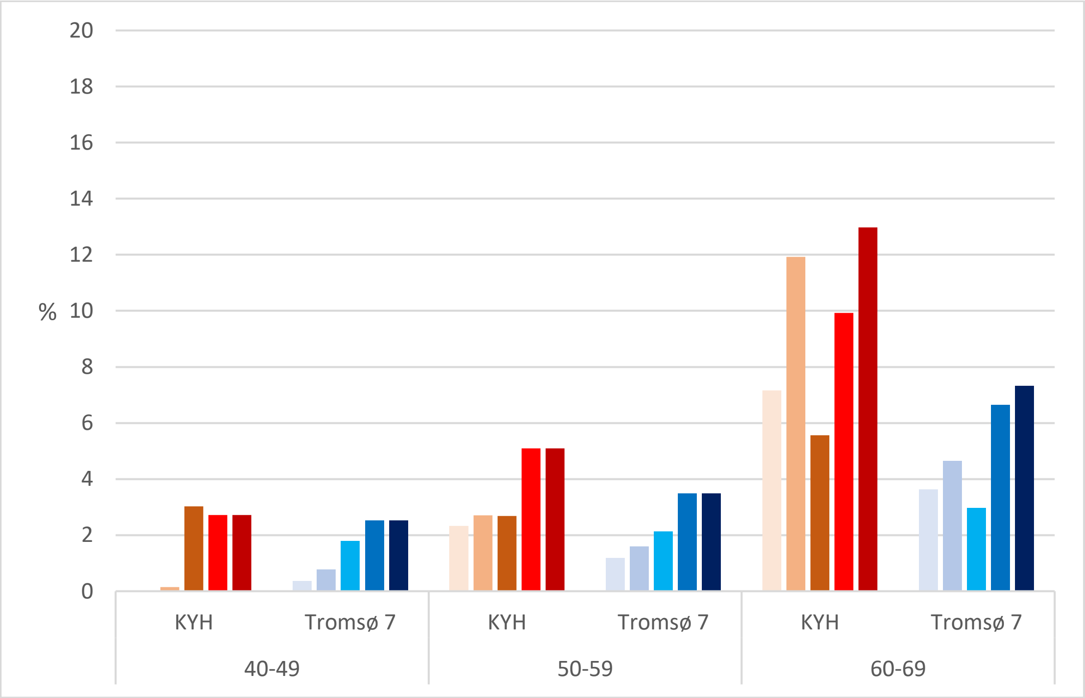

Supplementary Figure 2 Key

KYH

- eGFR<60 (CKD EPI creatinine equation)
- eGFR<60 (CKD EPI cystatin C- Creatinine equation)
- Albuminuria
- CKD (eGFR<60 CKD EPI creatinine equation and /or albuminuria
- CKD (eGFR<60 CKD EPI cystatin c- creatinine equation and/or albuminuria

Tromsø 7

- eGFR<60 (CKD EPI creatinine equation)
- eGFR<60 (CKD EPI cystatin C- Creatinine equation)
- Albuminuria
- CKD (eGFR<60 CKD EPI creatinine equation and /or albuminuria
- CKD (eGFR<60 CKD EPI cystatin c- creatinine equation and/or albuminuria
