# Supplementary material for: Comparing prevalence of chronic kidney disease and its risk factors between population-based surveys in Russia and Norway
Source: BMC Nephrol. 2022 Apr 14;23:145. doi: 10.1186/s12882-022-02738-2 (PMC9008943; doi:10.1186/s12882-022-02738-2)
Supplement: Supplementary file 3 — Additional file 3. [file 12882_2022_2738_MOESM3_ESM.docx]

**Supplementary Table 1. Predictors of reduced eGFR estimated from serum creatinine by Study (complete case analysis)**

|  | | Know Your Heart (n=3593) | | | Tromsø 7 (n=17053) | | | |
| --- | --- | --- | --- | --- | --- | --- | --- | --- |
|  |  | % | Age, sex and city adjusted OR* (95% CI) | Mutually adjusted OR*  (95% CI) | % | Age and sex adjusted OR* (95% CI) | | Mutually adjusted OR*  (95% CI) |
| Total sample | | 3.59 | - | - | 1.45 | - | | - |
| Age | 40-49 | 0.68 | 1.00 (ref) | 1.00 (ref) | 0.38 | 1.00 (ref) | | 1.00 (ref) |
|  | 50-59 | 2.37 | 3.46 (1.51, 7.96) | 2.63 (1.13, 6.11) | 0.82 | 2.15 | (1.32, 3.51) | 1.76 (1.07, 2.89) |
|  | 60-70 | 6.78 | 10.12 (4.67, 21.93) | 6.20 (2.77, 13.88) | 3.52 | 9.50 | (6.19, 14.58) | 5.68 (3.59, 8.96) |
|  | Test for trend |  | P<0.001 | P<0.001 |  | P<0.001 | | P<0.001 |
| Sex | Men | 3.57 | 1.00 (ref) | 1.00 (ref) | 1.56 | 1.00 | (ref) | 1.00 (ref) |
|  | Women | 3.62 | 1.02 (0.72, 1.47) | 0.88 (0.54, 1.42) | 1.33 | 1.22 | (0.94, 1.57) | 1.95 (1.31, 2.91) |
| Education | Low | 6.49 | 1.37 (0.79, 2.39) | 1.36 (0.78, 2.39) | 2.48 | 1.40 | (0.99, 1.97) | 1.36 (0.96, 1.92) |
|  | Middle | 3.83 | 1.00 (ref) | 1.00 (ref) | 1.26 | 1.00 | (ref) | 1.00 (ref) |
|  | Higher | 2.71 | 0.79 (0.53, 1.18) | 0.85 (0.56, 1.28) | 1.18 | 1.06 | (0.77, 1.46) | 1.15 (0.83, 1.60) |
|  | Test for trend |  | P=0.07 | P=0.14 |  | P=0.10 | | P=0.37 |
| Smoking status | Never smoker | 4.16 | 1.00 (ref) | 1.00 (ref) | 1.27 | 1.00 | (ref) | 1.00 (ref) |
|  | Ex-smoker | 2.23 | 0.53 (0.31, 0.92) | 0.48 (0.27, 0.83) | 1.73 | 1.03 | (0.78, 1.36) | 0.95 (0.72, 1.26) |
|  | Current smoker | 3.82 | 1.07 (0.67, 1.71) | 1.08 (0.67, 1.75) | 1.14 | 0.75 | (0.49, 1.14) | 0.73 (0.47, 1.12) |
| Body mass index | <18.5 | 0.0 | Perfect prediction | Perfect prediction | 2.35 | 2.04 (0.48, 8.64) | | 2.34 (0.54, 10.05) |
|  | 18.5-24.9 | 2.40 | 1.00 (ref) | 1.00 (ref) | 0.99 | 1.00 | (ref) | 1.00 (ref) |
|  | 25.0-29.9 | 3.26 | 1.05 (0.63, 1.75) | 0.98 (0.58, 1.66) | 1.52 | 1.54 | (1.11, 2.14) | 1.30 (0.93, 1.82) |
|  | 30.0-34.9 | 4.96 | 1.51 (0.89, 2.56) | 1.22 (0.70, 2.13) | 2.18 | 2.26 | (1.55, 3.26) | 1.56 (1.07, 2.29) |
|  | >35 | 5.30 | 1.62 (0.88, 3.01) | 1.21 (0.62, 2.33) | 1.17 | 1.38 | (0.73, 2.59) | 0.76 (0.39, 1.46) |
|  | Test for trend |  | P=0.03 | P=0.38 |  | P=0.001 | | P=0.98 |
| Waist Hip ratio | <0.9 | 2.61 | 1.00 (ref) | 1.00 (ref) | 1.22 | 1.00 | (ref) | 1.00 (ref) |
|  | >0.9 | 4.77 | 1.69 (1.12, 2.56) | 1.43 (0.93, 2.20) | 1.83 | 2.12 | (1.44, 3.11) | 1.63 (1.10, 2.43) |
| Albuminuria | No | 2.66 | 1.00 (ref) | 1.00 (ref) | 2.04 | 1.00 | (ref) | 1.00 (ref) |
|  | Yes | 15.49 | 5.09 (2.44, 10.61) | 5.14 (2.31, 11.45) | 11.59 | 6.40 | (3.95, 10.37) | 5.28 (3.07, 9.05) |
| Hypertension measured blood pressure/use of antihypertensives) | Normotensive | 1.43 | 1.00 (ref) | 1.00 (ref) | 0.75 | 1.00 | (ref) | 1.00 (ref) |
|  | Hypertensive  (treated and controlled) | 5.63 | 2.45 (1.40, 4.30) | 2.27 (1.27, 4.07) | 4.47 | 3.79 | (2.74, 5.24) | 3.41 (2.43, 4.78) |
|  | Hypertensive  (untreated) | 1.75 | 0.86 (0.39, 1.88) | 0.78 (0.35, 1.72) | 0.95 | 0.92 | (0.59, 1.43) | 0.86 (0.55, 1.35) |
|  | Hypertensive (treated, uncontrolled) | 6.18 | 2.51 (1.44, 4.38) | 2.26 (1.27, 4.02) | 4.15 | 3.20 | (2.22, 4.61) | 2.84 (1.94, 4.15) |
| Diabetes (self-report or HbA1c≥6.5% or use of medication) | No | 3.09 | 1.00 (ref) | 1.00 (ref) | 1.33 | 1.00 | (ref) | 1.00 (ref) |
|  | Yes | 6.56 | 1.49 (0.99, 2.26) | 1.15 (0.73, 1.78) | 3.64 | 2.10 | (1.44, 3.07) | 1.35 (0.90, 2.00) |

*Restricted to participants with data on all co-variates (except albuminuria)

Mutually adjusted models include all variables except low eGFR in the table with the exception BMI and waist-hip circumference not mutually adjusted for each other

**Supplementary Table 2. Association of risk factors with albuminuria by study using complete case analysis**

|  | | Know Your Heart (N=1614) | | | Tromsø 7 (N=5349) | | |
| --- | --- | --- | --- | --- | --- | --- | --- |
|  |  | % | Age, sex and city adjusted OR* (95% CI) | Mutually adjusted OR* (95% CI) | % | Age and sex adjusted OR (95% CI) | Mutually adjusted OR* (95% CI) |
| Total | | 4.40 |  |  | 3.87 |  |  |
| Age | 40-49 | 2.50 | 1.00 (ref) | 1.00 (ref) | 2.39 | 1.00 (ref) | 1.00 (ref) |
|  | 50-59 | 3.11 | 1.23 (0.57, 2.66) | 0.89 (0.40, 1.97) | 3.02 | 1.31 (0.78, 2.20) | 1.12 (0.66, 1.91) |
|  | 60-70 | 6.85 | 2.84 (1.45, 5.58) | 1.54 (0.73, 3.26) | 4.70 | 2.01 (1.33, 3.12) | 1.37 (0.85, 2.20) |
|  | Test for trend |  | P=0.001 | P=0.13 |  | P<0.001 | P=0.14 |
| Sex | Men | 5.15 | 1.00 (ref) | 1.00 (ref) | 5.54 | 1.00 (ref) | 1.00 (ref) |
|  | Women | 3.85 | 0.73 (0.45, 1.18) | 0.80 (0.42, 1.55) | 2.47 | 0.44 (0.30, 0.58) | 0.51 (0.33, 0.79) |
| Education | Low | 8.85 | 1.75 (0.83, 3.68) | 1.70 (0.79, 3.68) | 5.20 | 1.21 (0.85, 1.73) | 1.10 (0.76, 1.58) |
|  | Middle | 4.39 | 1.00 (ref) | 1.00 (ref) | 4.11 | 1.00 (ref) | 1.00 (ref) |
|  | Higher | 3.64 | 0.87 (0.51, 1.49) | 1.01 (0.59, 1.76) | 3.06 | 0.77 (0.55, 1.08) | 0.92 (0.65, 1.30) |
|  | Test for trend |  | P=0.14 | P=0.38 |  | P=0.009 | P=0.34 |
| Smoking status | Never smoker | 4.02 | 1.00 (ref) | 1.00 (ref) | 3.20 | 1.00 (ref) | 1.00 (ref) |
|  | Ex-smoker | 4.55 | 1.14 (0.60, 2.16) | 0.98 (0.51, 1.90) | 3.41 | 1.02 (0.73, 1.42) | 0.91 (0.65, 1.27) |
|  | Current smoker | 5.23 | 1.44 (0.74, 2.78) | 1.39 (0.70, 2.75) | 7.13 | 2.34 (1.61, 3.39) | 2.17 (1.47, 3.22) |
| Body mass index | <18.5 | 0.00 | Perfect prediction | Perfect prediction | 15.15 | 7.86 (2.85, 21.69) | 5.98 (2.11, 16.99) |
|  | 18.5-24.9 | 3.23 | 1.00 (ref) | 1.00 (ref) | 2.75 | 1.00 (ref) | 1.00 (ref) |
|  | 25.0-29.9 | 3.61 | 0.94 (0.48, 1.85) | 0.79 (0.39, 1.59) | 3.45 | 1.04 (0.72, 1.51) | 0.91 (0.63, 1.34) |
|  | 30.0-34.9 | 4.57 | 1.30 (0.63, 2.67) | 0.91 (0.42, 1.96) | 4.91 | 1.52 (1.00, 2.32) | 1.12 (0.72, 1.74) |
|  | >35 | 10.90 | 3.39 (1.61, 7.13) | 2.20 (0.96, 5.00) | 9.03 | 3.36 (2.05, 5.52) | 1.96 (1.15, 3.36) |
|  | Test for trend |  | P=0.002 | P=0.06 |  | P<0.001 | P=0.14 |
| Waist Hip ratio | <0.9 | 3.11 | 1.00 (ref) | 1.00 (ref) | 2.48 | 1.00 (ref) | 1.00 (ref) |
|  | >0.9 | 5.91 | 1.75 (0.99, 3.10) | 1.23 (0.68, 2.24) | 6.06 | 1.77 (1.15, 2.70) | 1.23 (0.78, 1.94) |
| Reduced eGFR based on serum creatinine | No | 3.84 | 1.00 (ref) | 1.00 (ref) | 3.51 | 1.00 (ref) | 1.00 (ref) |
|  | Yes | 21.15 | 5.08 (2.44, 10.59) | 4.51 (2.01, 10.11) | 18.60 | 6.32 (3.91, 10.24) | 5.23 (3.13, 8.75) |
| Hypertension (measured blood pressure/use of antihypertensives) | Normotensive | 1.67 | 1.00 (ref) | 1.00 (ref) | 2.13 | 1.00 (ref) | 1.00 (ref) |
|  | Hypertensive  (treated and controlled) | 3.61 | 1.82 (0.78, 4.22) | 1.45 (0.60, 3.52) | 7.31 | 3.08 (2.10, 4.51) | 2.50 (1.66, 3.77) |
|  | Hypertensive  (untreated) | 3.86 | 2.02 (0.79, 5.16) | 1.84 (0.71, 4.76) | 4.29 | 1.80 (1.21, 2.69) | 1.68 (1.12, 2.52) |
|  | Hypertensive (treated, uncontrolled) | 10.11 | 5.29 (2.47, 11.30) | 4.51 (2.03, 9.98) | 7.27 | 2.96 (1.95, 4.49) | 2.46 (1.58, 3.84) |
| Diabetes  (self-report or HbA1c≥6.5% or use of medication) | No | 3.54 | 1.00 (ref) | 1.00 (ref) | 3.28 | 1.00 (ref) | 1.00 (ref) |
|  | Yes | 9.57 | 2.38 (1.38, 4.09) | 1.70 (0.94, 3.08) | 12.18 | 3.56 (2.48, 5.11) | 2.37 (1.60, 3.49) |

*Restricted to participants with data on all co-variates (except eGFR)

Mutually adjusted models include all variables except low eGFR in the table with the exceptions body mass index and waist-hip circumference not mutually adjusted for each other

**Supplementary Table 3: Association between Study (Know Your Heart/Tromsø 7) and reduced eGFR and albuminuria adjusting for known risk factors (complete case analysis)**

|  | Reduced eGFR <60ml/min  /1.73m2 (%) (n=20,606) | | Elevated albuminuria ≥30mg/g albumin/creatinine (n=6,941) | |
| --- | --- | --- | --- | --- |
|  | OR (95% CI) | P value | OR (95% CI) | P value |
| + Age +Sex | 2.10 (1.69, 2.62) | <0.001 | 1.32 (1.00, 1.76) | 0.05 |
| + Age + sex+ education | 2.21 (1.75, 2.78) | <0.001 | 1.35 (1.01, 1.82) | 0.04 |
| + Age + sex + smoking status | 2.07 (1.65, 2.59) | <0.001 | 1.24 (0.93, 1.66) | 0.14 |
| + Age + sex + body mass index + waist hip ratio | 1.88 (1.50, 2.36) | <0.001 | 1.17 (0.88, 1.57) | 0.28 |
| + Age + sex + systolic blood pressure | 2.05 (1.64, 2.56) | <0.001 | 1.20 (0.90, 1.60) | 0.21 |
| + Age + sex + diastolic blood pressure | 2.01 (1.59, 2.55) | <0.001 | 1.09 (0.81, 1.48) | 0.56 |
| + Age + sex + systolic blood pressure + diastolic blood pressure | 2.06 (1.62, 2.61) | <0.001 | 1.20 (0.88, 1.63) | 0.25 |
| + Age +sex + antihypertensive medication | 1.50 (1.19, 1.89) | 0.001 | 1.04 (0.77, 1.39) | 0.81 |
| + Age + sex + diabetes | 1.71 (1.29, 2.27) | <0.001 | 1.14 (0.85, 1.52) | 0.39 |
| + Age and sex + smoking+ body mass index + waist hip ratio + diastolic blood pressure + diabetes | 1.73 (1.35, 2.23) | <0.001 | 0.84 (0.62, 1.15) | 0.28 |
| + Age and sex + smoking+ body mass index + waist hip ratio+ systolic blood pressure + diastolic blood pressure + diabetes | 1.76 (1.36, 2.27) | <0.001 | 0.91 (0.66, 1.25) | 0.57 |
| + Age and sex + smoking+ body mass index + waist hip ratio+ systolic blood pressure + diastolic blood pressure + diabetes + antihypertensive medication | 1.38 (1.06, 1.78) | 0.02 | 0.78 (0.56, 1.07) | 0.13 |

*Restricted to participants with complete data on all co-variates
