# Supplementary figures and images for: Comparing prevalence of chronic kidney disease and its risk factors between population-based surveys in Russia and Norway
Source: BMC Nephrol. 2022 Apr 14;23:145. doi: 10.1186/s12882-022-02738-2 (PMC9008943; doi:10.1186/s12882-022-02738-2)

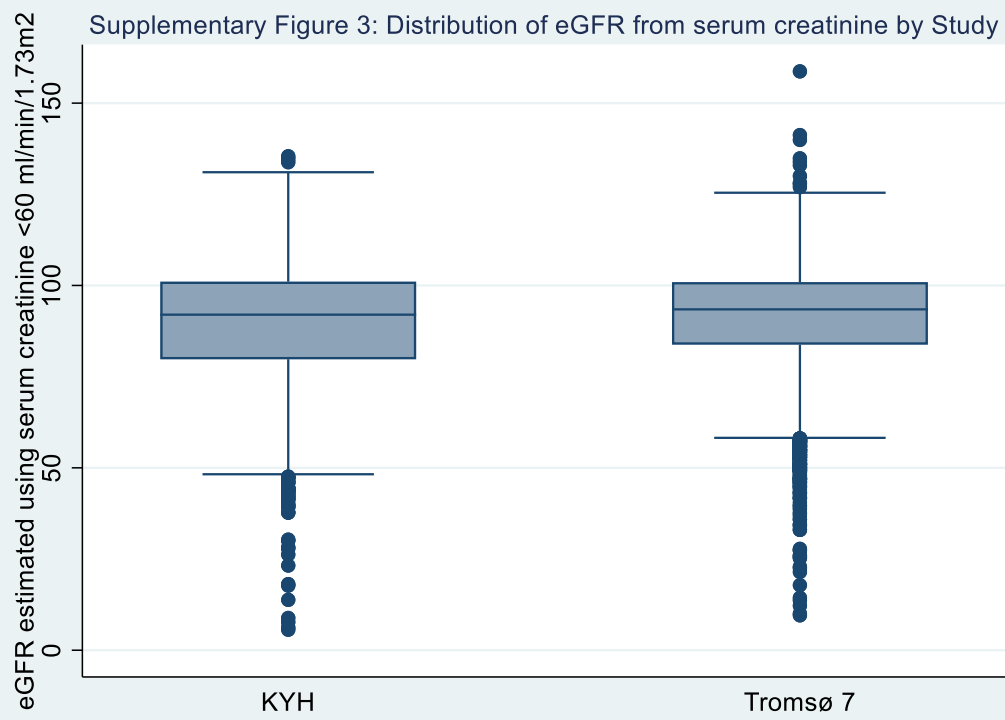

Supplement: Supplementary file 4 — Additional file 4. [file 12882_2022_2738_MOESM4_ESM.pdf]

Supplementary Figure 4. Distribution of urinary albumin to creatinine ratio by Study

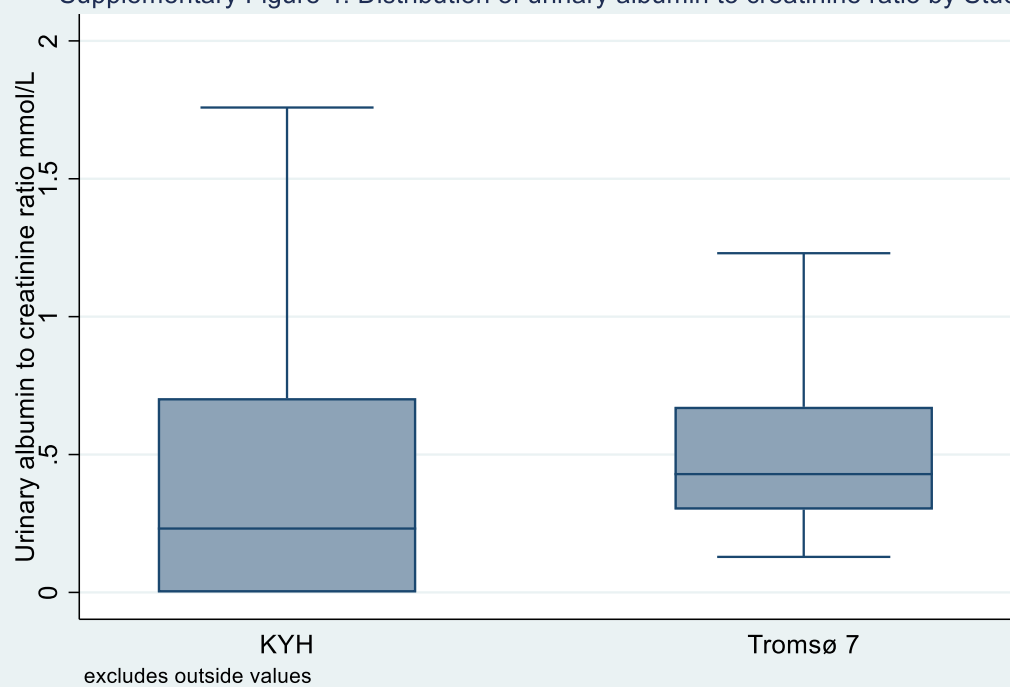

Supplement: Supplementary file 5 — Additional file 5. [file 12882_2022_2738_MOESM5_ESM.pdf]
